# Proteomic Analysis of the Salt-Responsive Leaf and Root Proteins in the Anticancer Plant *Andrographis paniculata* Nees

**DOI:** 10.1371/journal.pone.0112907

**Published:** 2014-11-25

**Authors:** Daryush Talei, Alireza Valdiani, Mohd Yusop Rafii, Mahmood Maziah

**Affiliations:** 1 Medicinal Plants Research Center, Shahed University, Tehran, Iran; 2 Department of Cell and Molecular Biology, Faculty of Biotechnology and Biomolecular Sciences, Universiti Putra Malaysia, UPM Serdang, Selangor DE, Malaysia; 3 Department of Biochemistry, Faculty of Biotechnology and Biomolecular Sciences, Universiti Putra Malaysia, UPM Serdang, Selangor DE, Malaysia; 4 Institute of Tropical Agriculture, Universiti Putra Malaysia, UPM Serdang, Selangor DE, Malaysia; 5 Institute of Bioscience, Universiti Putra Malaysia, UPM Serdang, Selangor DE, Malaysia; National Taiwan University, Taiwan

## Abstract

Separation of proteins based on the physicochemical properties with different molecular weight and isoelectric points would be more accurate. In the current research, the 45-day-old seedlings were treated with 0 (control) and 12 dS m^−1^ of sodium chloride in the hydroponic system. After 15 days of salt exposure, the total protein of the fresh leaves and roots was extracted and analyzed using two-dimensional electrophoresis system (2-DE). The analysis led to the detection of 32 induced proteins (19 proteins in leaf and 13 proteins in the root) as well as 12 upregulated proteins (four proteins in leaf and eight proteins in the root) in the salt-treated plants. Of the 44 detected proteins, 12 were sequenced, and three of them matched with superoxide dismutase, ascorbate peroxidase and ribulose-1, 5-bisphosphate oxygenase whereas the rest remained unknown. The three known proteins associate with plants response to environmental stresses and could represent the general stress proteins in the present study too. In addition, the proteomic feedback of different accessions of *A. paniculata* to salt stress can potentially be used to breed salt-tolerant varieties of the herb.

## Introduction

*Andrographis paniculata* is a medicinal herb from the family Acanthaceae. The plant extract contains diterpene compounds with a broad scope of pharmaceutical properties such as anticancer, antibacterial, antivirus and anti-hepatitis [1]. Salinity stress alters various biochemical and physiological responses in plants and causes adverse effects on various physiological processes such as photosynthesis, growth and development [2], [3]. From a molecular perspective, salt stress comprises many factors, including oxidative stress, osmotic stress, ion stress, nutritional imbalance or a combination of these factors [4], [5].

Plants produce antioxidants and protective enzymes such as superoxide dismutase, catalase, peroxidase, glutathione reductase, polyphenol oxidase that scavenge the reactive oxygens or prevent their formation, to reduce oxidative damage [6]. In response to salinity, plants accumulate organic compounds (proline, glycine-betaine, malate, and polyol) in the cytoplasm, which function as osmolytes preventing conformational changes of macromolecules especially proteins [7].

Under certain level of salinity, some of those major macromolecules involved with various cellular processes are prone to the conformational changes of their native structures affecting the number and level of proteomes in the affected tissues. These changes should be monitored through profiling the proteomes of the affected tissues/organs, after being exposed to salt, regularly. The changes could include protein modifications, proteolysis, subcellular localizations, and interaction with other proteins [8]. Many proteins undergo post-translational modifications including phosphorylation, which play an important role in subcellular localization [8], [9].

With respect to salinity, several salt-responsive proteins have been reported in rice (*Oryza sativa*) [10], [11], *Arabidopsis thaliana* [12], and wheat (*Triticum aestivum*) [13]. These proteins associate with major cellular processes including photosynthesis, photorespiration, metabolic regulation, signal transduction, control of ion channels, oxidative stress defence and protein folding.

An enormous lack of information related to the salt responsive proteins of *A. paniculata* is tangible. Therefore, the present study emphasized on the analysis and identification of the salt stress responsive proteins of this herb under salinity stress conditions using two-dimensional gel electrophoresis.

## Results and Discussion

### Analysis of protein spots separated by 2-DE PAGE using the PDQuest software

**Figure 1:**
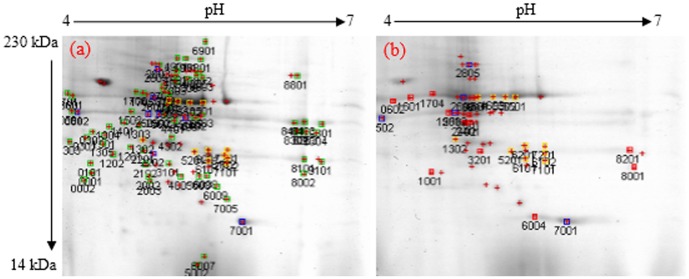
The super composite images of 2-DE gels representing the repressed leaf proteins of *A. paniculata* in control (a) and salt-treated samples (b).

**Figure 2:**
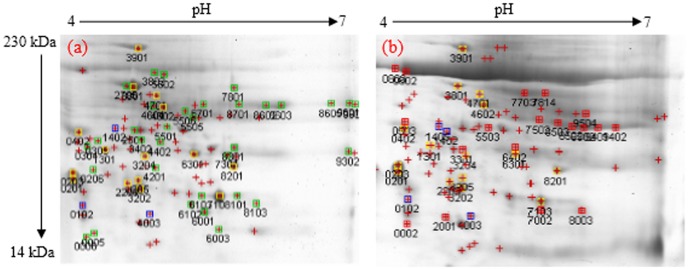
The super composite images of 2-DE gels representing the repressed root proteins of *A. paniculata* in control (a) and salt-treated samples (b).

A total of 162 induced upregulated and downregulated proteins were detected in the control and salt-treated leaf and root samples of *A. paniculata* seedlings. Dissimilar expression patterns of the expressed proteins were observed in both of the control and salt-treated plants. Figures 1 and 2 represent the typical super composite images of the leaf and root samples. The analysis revealed a total of 15 upregulated and four downregulated leaf proteins in the control and salt-treated samples, respectively as shown in Table 1. Furthermore, 12 upregulated and eight downregulated root proteins were detected in the control and salt-treated samples, respectively (Table 1). Comparison of the total number of representing proteins (repressed, induced, upregulated and downregulated proteins) in the control and salt-treated samples using independent samples t-test showed non-significant differences in the studied organs such as leaves, roots, as well as the whole plant (Table 2).

**Table 1:** The number of unique/common expressed proteins of the leaf and root samples of *A. paniculata* under control and salinity condition.

Protein types	Leaf		Root	
	Control	Salt-treated	Control	Salt-treated
Repressed	85	71	77	17
Induced	33	19	63	13
Upregulated	15	4	12	8
Downregulated	4	15	8	12

Repressed proteins: The protein spots absent in the salt-treated samples and present in the controls, Induced proteins: The protein spots present in the salt-treated samples and absent in the controls, Downregulated proteins: The protein spots that their size and intensity was decreased in the salt-treated samples as compared to the control samples, Upregulated proteins: The protein spots that their size and intensity was increased in the salt-treated samples as compared to the control samples.

**Table 2:** Comparison of the total number of representing proteins (repressed, induced, upregulated and downregulated proteins) in the control and salt-treated samples using independent samples t-test.

Organ	Group	N	Mean	SD	T	df	Sig.	SE Difference
Leaf	Control	4	34.25	35.88	0.3^ns^	6	0.774	23.34
	Salt-treated	4	27.25	29.85				
Root	Control	4	40.00	35.15	1.56^ns^	6	0.171	17.67
	Salt-treated	4	12.50	3.70				
Whole plant	Control	8	37.13	33.03	1.24^ns^	14	0.243	13.88
	Salt-treated	8	19.88	21.21				

N: Number of samples, SD: Standard deviation, T: T-test value, df: Degree of freedom, Sig: Statistical significance, SE: Standard error, ns: Non-significant.

Several proteins were differentially expressed (repressed, induced, upregulated and downregulated) in the seedlings exposed to high salinity (12 dS m^−1^). Previous studies on different plant species under salinity stress have discovered a number of differently expressed proteins. These proteins link to several functional categories such as transcription factors, solute accumulation enzymes and stress tolerance proteins e.g. superoxide dismutase (SOD) [14]–[18], ascorbate peroxidases [19]–[22], transcription factors [23], [24], protein kinases [25], protein phosphatases [26], ATP generation [24] and calcium related signalling molecules [27]. As a matter of fact, the role of the expressed proteins of *A. paniculata* remained unidentified in this study. For this reason, further functional analyses of the proteins will be useful to identify their roles in salt tolerance mechanism, in the next studies.

As was anticipated, the protein expression patterns of the leaves and roots were different in the control and slat-treated accessions. In this study, the protein was considered as “induced”, when it was only present in the treated samples, but not in the control. The number of the unique proteins of the leaf and root samples is available in Table 1. There were altogether 19 and 13 induced protein spots in the treated leaf and root samples, respectively, four of which were “commonly” induced in both of the control and salt-treated samples. The uniquely induced proteins might be related to the specific genes involved with the plant's response to salinity stress whereas the “commonly” induced proteins could represent a general stress response at various degrees of salt stress. Apart from the induced proteins, the software also facilitated the detection of repressed protein, i.e. the protein spots which are absent on the salt-treated samples, but are present in the control samples. Table 3 represents the number of the repressed proteins.

**Table 3:** List of the induced and upregulated protein spots detected in the salt-treated leaf and root samples of *A. paniculata* at seedling stage.

Salt-treated leaf				Salt-treated root			
Spot No.	pI (pH)	Molecular weight (kDa)	Regulation trend	Spot No.	pI (pH)	Molecular weight (kDa)	Regulation trend
L-0002	4.35	21.28	**	R-0602	4.38	61.33	**
L-0503	4.48	42.3	**	R-1001	4.65	28.42	**
L-0802	4.38	78.41	**	R-1601	4.42	63.4	**
L-0803	4.44	72.47	**	R-1704	4.78	66.27	**
L-2001	4.78	18.8	**	R-1501	4.85	50.14	**
L-3301	4.95	35.74	**	R-2402	4.98	47.83	**
L-5503	5.28	41.37	**	R-2401	5.12	46.78	**
L-6402	5.35	38.48	**	R-2501	5.04	48.61	**
L-7002	5.65	20.72	**	R-3201	5.25	38.26	**
L-7502	5.62	47.31	**	R-3601	5.14	63.73	**
L-7703	5.45	65.42	**	R-6004	5.52	21.64	**
L-7814	5.65	64.73	**	R-8001	6.41	32.72	**
L-8003	6.15	20.4	**	R-8201	6.35	38.47	**
L-8502	5.82	37.42	**	R-0502	4.28	47.23	*
L-8503	5.98	32.75	**	R-2202	5.05	37.82	*
L-9401	6.15	31.34	**	R-2502	4.12	46.88	*
L-9402	6.22	30.87	**	R-2503	4.92	48.74	*
L-9502	6.12	29.83	**	R-2602	5.04	56.73	*
L-9504	6.18	48.54	**	R-2805	5.11	87.82	*
L-0102	4.46	24.84	*	R-5403	5.34	47.36	*
L-1402	4.82	41.83	*	R-7001	5.87	19.62	*
L-1405	4.74	43.18	*	-	-	-	-
L-4003	5.12	18.35	*	-	-	-	-

**Induced proteins and * Upregulated proteins based on two fold. pI: Isoelectric point.

Salt stress affects almost every aspect of the plant physiology and metabolism. Since, root is the primary target of many abiotic stresses; therefore, the current research considered it as the first responding organ of the plant to the salt stress. The response of *A. paniculata* roots to salinity stress was more complex, in which the number of the induced and upregulated proteins in the root were higher than the number of these proteins in the leaf samples. Almost all of the induced and upregulated proteins in the leaf and root samples were different in terms of the estimated isoelectric points (pI), and molecular weights (MW) with the exception of three detected identical proteins (Table 3). The higher number of the repressed proteins in the root compared to the leaf in response to salt treatment indicated that roots are more prone to the effects of salt stress and are severely affected by salinity. The independent samples t-test results showed non-significant differences between the induced proteins of the salt-treated leaf and root samples, as well as between the upregulated proteins of the salt-treated leaf and root samples in terms of the molecular weights (Table 4). However, the average molecular weights of the induced and upregulated proteins in the salt-treated root were higher than the leaf (Table 4).

**Table 4:** Comparison of the induced and upregulated proteins in the salt-treated leaf and root samples of *A. paniculata* on the basis of molecular weight using independent samples t-test.

Protein	Group	N	Mean	SD	T	Df	Sig.	SE Difference
Induced	Leaf	19	40.96	17.95	0.967^ns^	30	0.341	5.98
	Root	13	46.74	14.37				
Upregulated	Leaf	4	32.05	12.37	1.594^ns^	10	0.142	10.65
	Root	8	49.03	19.14				

N: Number of samples, SD: Standard deviation, T: T-test value, df: Degree of freedom, Sig: Statistical significance, SE: Standard error, ns: Non-significant.

Overall, the total number of expressed proteins in the salt-treated leaves and roots was decreased, which might reflect the adverse effects of salinity on growth and development of the plant. Many cellular and metabolic processes of plants are known to be affected by salinity, including the reductions in the stromal volume of chloroplast, generation of reactive oxygen species (ROS), photosynthesis, respiration, biosynthesis of protein, nucleic acid, lipids, and pigments [28]. Reportedly, in some plant species such as *Oryza sativa* [29] and *Bruguiera parviflora* [30], decrease in the number of proteins happened in response to salinity.

**Figure 3:**
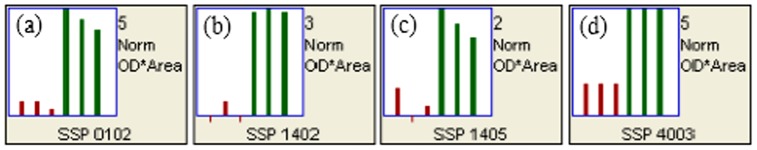
Histogram representing the four upregulated leaf proteins of *A. paniculata* in the salt-treated plants. The red color refers to the quantity of leaf proteins in control samples (downregulated) and the green color shows the quantity of leaf proteins in salt-treated samples (upregulated). Spot No. 0102 (a), spot No. 1402 (b), spot No. 1405 (c) and spot No. 4003 (d).

**Figure 4:**
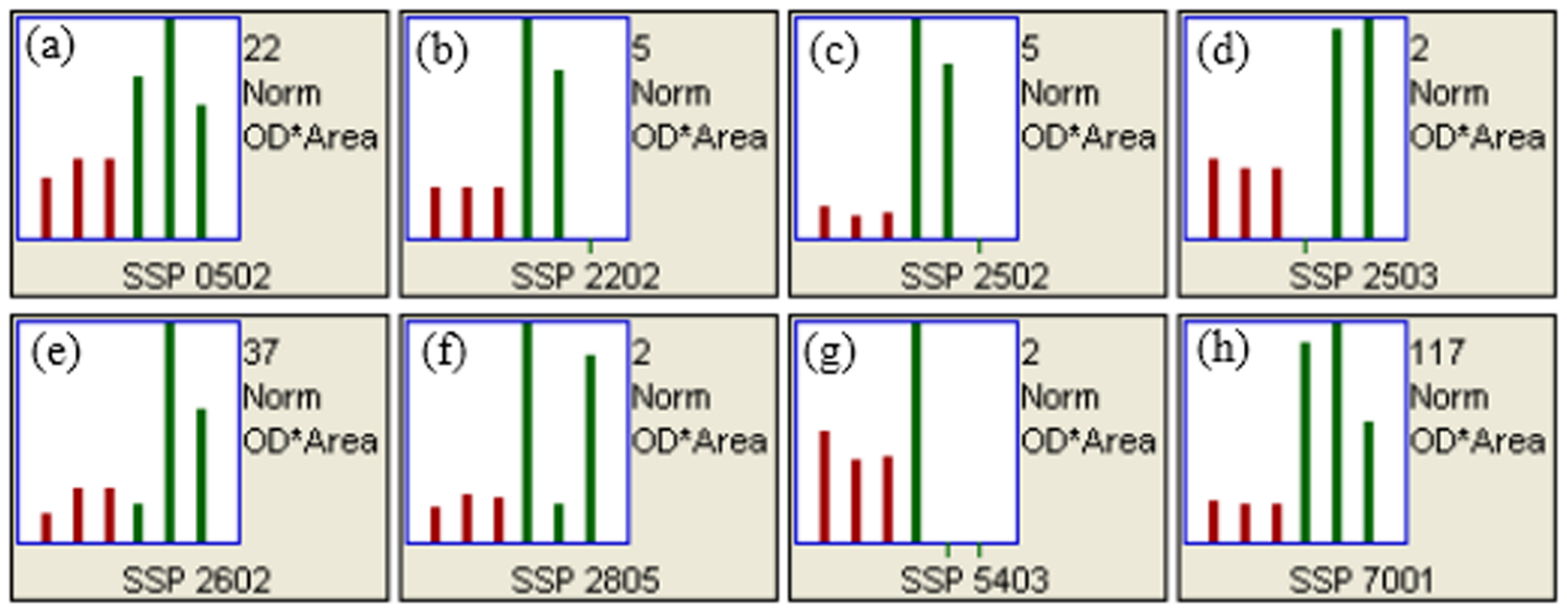
Histogram representing the eight upregulated root proteins of *A. paniculata* in the salt-treated plants. The red color refers to the quantity of root proteins in control samples (downregulated) and the green color shows the quantity of root proteins in salt-treated samples (upregulated). Spot No. 0502 (a), spot No. 2202 (b), spot No. 2502 (c) and spot No. 2503 (d), spot No. 2602 (e), spot No. 2805 (f) and spot No. 4503 (g) and spot No. 7001 (h).

The magnitude of upregulated proteins in the root and leaf samples of the treated plants varied from spot to spot. The magnitude of expression or intensity reflects the level of induction of the responsive proteins, which in turn indicates the pathways responding the salt stress. The expression intensities of the four upregulated proteins varied in the leaves and roots of the treated plants as shown by the histograms in Figure 3 and 4, respectively.

Unfortunately, the whole genome of the plant is still unsequenced; hence, the online search of the database with emphasis on molecular weight and isoelectric point information sole, yielded little success, as experienced during this study. Consequently, identification of the proteins became impossible in this manner.

### Classification of the Identified Functional Proteins

In total, 12 induced, upregulated and downregulated leaf and root proteins of the plant were identified using mass spectrometry technique. Tables 5 and 6 show the identity of the protein spots as determined by the peptide mass fingerprinting method and Table 7 demonstrates the math rate and correlation coefficient of protein spots among three replicate of the leaf and root samples at control and salinity levels using the PDQuest software.

**Table 5:** Identification of the 12 induced and upregulated protein spots in the salt-treated leaf and root samples of *A. paniculata* as determined by 2-DE analysis, MALDI-TOF-MS, and MASCOT analysis.

Spot No.	Spot ID	Regulation trend	Protein name	Sequence	Mass (Da)	Mascot score	MPN
L-0802	167858	**	Ribulose-1,5-bisphosphate carboxylase/oxygenase	K.DTDILAAFR.V	49310	427	6(6)
L-0402	167859	***	(Unknown protein)	VNFPEVPR	Unknown	Unknown	Unknown
L-4003	167860	*	Putative uncharacterized protein	K. ALFSQITTRF.-	35649	432	3(3)
L-3202	167861	***	Putative uncharacterized protein	R.SVDETLR.T	30801	545	5(4)
L-7002	167862	**	Superoxide dismutase (Cu-Zn)	K.GGHELSLSTGNAGGR.L	20883	372	3(3)
L-2001	167863	**	(Unknown protein)	LITPEGEK	Unknown	Unknown	Unknown
L-5503	167867	**	Os04g0416400 protein (Fragment)	R.GLFIIDKEGVIQHSTINNLAIGR.S	15378	57	1(1)
L-8003	167869	**	Putative uncharacterized protein	R.GQQLLDTFR.V	12438	44	1(1)
R-6501	167864	*	Q0VYC8|Peroxidase 1	R.SPNVFDNR.Y	39905	66	1(1)
R-7001	167865	*	Superoxide dismutase (Cu-Zn)	K.EHGAPEDETR.H	15217	262	3(2)
R-2401	167866	**	Putative uncharacterized protein	K.TLVFQFSVK.H	49129	300	4(4)
R-2501	167857	**	Putative salt-inducible protein	RMTEFGKIGMLMTLFKQLK	18737	372	4(4)

***Downregulated proteins, ** Induced proteins and * Upregulated proteins. MPN: Matched peptide number. Mascot score: Total ion score for the entire protein and for ions complemented by 100% of the confidence index (C.I).

**Table 6:** Physicochemical characteristics of the 12 induced and upregulated protein spots in the salt-treated leaf and root samples of *A. paniculata* as determined by 2-DE analysis, MALDI-TOF-MS, and MASCOT analysis.

Spot No.	Spot ID	TMM (kDa)	EMM (kDa)	EpI (pH)	TpI (pH)	Protein coverage (%)	Function	Taxonomy identifier
L-0802	167858	4.93	1.02	6.43	4.38	18	Salt-inducible protein	883475 [*Barleria* sp.]
L-0402	167859	-	0.96	-	4.26	-	Unknown	NSHR [*Populus trichocarpa*]
L-4003	167860	3.56	1.18	8.91	5.12	5	Probable salt-inducible protein	3847 [*Glycine max*]
L-3202	167861	3.08	0.82	8.56	4.82	22	Probable salt-inducible protein	3332 [*Picea sitchensis*]
L-7002	167862	2.09	1.41	5.45	5.65	15	Antioxidant (salt induced protein)	4577 [*Zea mays*]
L-2001	167863	-	0.88	-	4.78	Unknown	Unknown	NSHR [*Oryza punctate*]
L-5503	167867	1.54	2.51	5.87	5.28	14	Antioxidant	39947 [*Oryza sativa* subsp. *Japonica*]
L-8003	167869	1.24	1.08	10.11	6.15	-	Probable salt-inducible protein	29760 [*Vitis vinifera*]
R-6501	167864	3.99	0.95	7.63	6.02	2	Detoxification (salt induced protein)	4058 [*Catharanthus roseus*]
R-7001	167865	1.52	1.14	5.45	5.87	21	Antioxidant(salt induced protein)	3880 [*Medicago truncatula*]
R-2401	167866	4.91	1.07	4.45	5.12	8	Probable salt-inducible protein	81972 [*Arabidopsis lyrata* subsp. *Lyrata*]
R-2501	167857	1.87	1.47	5.87	5.04	15	Probable salt-inducible protein	NSHR [*Arabidopsis*]

EpI: Experimental isoelectric point. TpI: Theoretical isoelectric point. TMM: Theoretical molecular mass, EMM: Experimental molecular mass, Protein coverage: Percentage of protein coverage. Mr: The nominal mass of an ion or molecule that is calculated using the mass of the most abundant isotope of each element rounded to the nearest integer (Da). NSHR: No significant hits to report.

**Table 7:** The results of automath settings of protein spots in the leaf and root sample of *Andrographis paniculata*.

Replicates	Gel name	Spot No.	Matched	Match rate 1 (%)	Match rate 2 (%)	Correlation coefficient
1	LC1	96	85	88	57	1.00
2	LC1	96	87	90	59	0.98
3	LC1	96	87	90	59	0.96
1	LC4	95	71	74	48	0.50
2	LC4	95	71	74	48	0.59
3	LC4	95	71	74	48	0.60
1	RC1	114	112	98	82	0.92
2	RC1	114	114	100	84	1.00
3	RC1	114	112	98	82	0.99
1	RC4	57	47	82	34	0.50
2	RC4	57	50	87	37	0.58
3	RC4	58	49	84	36	0.47

LC1: Leaf sample at control level, LC4: Leaf sample at 12 dS m^−1^ salinity level, RC1: Root sample at control level, RC4: Root sample at 12 dS m^−1^ salinity level. High correlation coefficients indicate high degree of confidence of the matched spots.

The sequencing result revealed the two salt stress responsive enzymes known as superoxide dismutase (SOD) and ascorbate peroxides (APX), which have a central role in oxidative plant defense mechanisms. Plants possess a number of antioxidant enzymes such as SOD, APX and glutathione reductase (GR) for protecting themselves against the injurious effects of reactive oxygen species (ROS) [31]. Salinity stress induces production of ROS in plants. Major scavenging mechanisms of ROS connect to the production of SOD and APX enzymes. Antioxidant enzymes are essential elements in the defense mechanisms. Therefore, the inability of plants in tolerating salt stress is attributed to a decrease in the activity of the mentioned antioxidant enzymes [16]. In accordance with similar studies SOD and APX were induced, under salinity condition in the present experiment as well. Increment in the activity of SOD correlates to the increased level of protection against the damages associated with oxidative stress [32], [33]. In agreement with the present results, the increased activity of antioxidant enzymes under salt stress has been reported in some salt tolerant plants such as cotton [34], cucumber [35], pea [36], *Solanum tuberosum* [37], *Pisum sativum* [38], wild tomato species *Lycopersicon pennellii* [16], rice (*Oryza sativa*) [17], and soybean (*Glycine max*) [39].

Ascorbate peroxidase is a hydrogen peroxide-scavenging enzyme that is essential for plants to protect chloroplasts and the other cell components from damages driven by hydrogen peroxide and its hydroxyl radicals [40]. The enzyme can improve the levels of salt and drought tolerance by scavenging H_2_O_2_ that is unreachable for catalase because of their high similarity with H_2_O_2_ and also their presence in different subcellular locations [41]. Analysis of the APX activity in some plant species such as *Arabidopsis thaliana* [21], *Oryza sativa*, *Hordeum vulgare*, *Triticum aestivum*, *Lolium perenne*, and *Zea mays* showed a significant high resolution [14], [19], [20].

Another upregulated enzyme was ribulose-1, 5-bisphosphate oxygenase also known as Rubisco. This enzyme is a critical factor in the process of photosynthesis. During salinity stress, the available water capacity (AWC) is limited in soil, and since water is extremely crucial for photosynthesis, the photosynthetic rate of the plants should be lower under such a condition compared to the standard situation. However, leaf analysis showed that Rubisco was upregulated in the salt-treated plants, but it is unclear whether the increment of Rubisco was due to an increase in photosynthetic activity of the plant or caused by another reason. Nevertheless, according to the previous findings, Rubisco is also associated with proline accumulation [42] that the same happened to the salt-treated plants of the present study.

The intensive response of the plants to salinity in the root compared to leaf, suggesting roots as a principal organ for mediating the plant's response to salinity. Most of the induced and upregulated proteins differed in their estimated isoelectric points (pI) and molecular weights (MW) in the leaf and root of the salt-treated plants, which might suggest that different metabolic pathways be functional in the leaf and root of the plant during the stress. A few of these responsive proteins were identified, and their functions are related to plant responses to the stress conditions.

Despite the lack of applicable information on the genome of *A. paniculata*, protein profiling of this plant will serve as an introduction to its proteomics. The current results can also provide the researchers with a reliable background based on protein function in different parts of *A. paniculata* and their effects on biological phenomena of the herb. However, a pure focus on the performance of the proteins of this plant species will release a huge volume of useful information in the future. Undoubtedly, two dimensional proteomics analysis is one of the most efficient technologies to acquire cutting-edge knowledge on the genetics and physiology of plants, including *A. paniculata*.

The systematic proteomic analysis of *A. paniculata* under salinity stress led to the identification of upregulated, downregulated, repressed and induced proteins in control and salt-treated plants. The combination of two-dimensional electrophoresis (2-DE) and mass spectrometry revealed the plasticity of protein expression during salinity stress. These proteins belong to several functional groups such as osmotic stress related proteins and general stress proteins. Induction of some of these proteins was observed in the roots of salt-treated *A. paniculata* plants, and this might be connected to the plant response to salt stress. The proteins might either be directly involved in the protection against environmental stresses or play a role in stress regulation and signal transduction. Overexpression of such proteins should make the plant more resistant to the stress if the limiting factor in resistance is either the timing or level of expression of the gene in question. Functional testing by overexpression could initiate developing new plant varieties with a higher capability of resistance.

### Application of proteomic data in *A. paniculata* breeding

As a promising horizon in the breeding of *A. paniculata*, intraspecific hybridization has led to considerable results by increment in the contents of anticancer diterpenes such as andrographolide (AG) [43], and neoandrographolide (NAG), and 14-deoxy-11,12-didehydroandrographolide (DDAG) [44]. Furthermore, recently conducted genetic investigations have revealed that morphological- and DNA-based genetic distance could affect the heterosis of phytochemical as well as Agro-morphological characteristics in *A. paniculata* [45], [46]. In this regard, proteomic data can be effectively employed for breeding purposes [47] and assessing the impact of outcrossing on diversification of protein pattern of the herb through the recombinant proteins. A prospective study should tend to quantify the proteomic data and link them to the breeding approaches of this precious herb, in the future.

## Conclusion

Proteins are the final production of genes and one of the basic adaptive strategies in plants under salinity stress. Overexpression and identification of the salt-induced proteins can be potentially used in genetic engineering of *A. paniculata* and improving its salt tolerance capacity. Since, cells have the ability to monitor the severity of stress and the degree of stress-induced damage, the identified proteins in this study could represent a so-called “stress proteome” in response to salinity for *A. paniculata*.

## Materials and Methods

### Chemicals and Reagents

All the chemicals, including HEPES [4-(2-hydroxyethyl)-1-piperazineethanesulfonic acid], thiourea, bovine serum albumin (BSA), iodoacetamide, and ethylenediaminetetraacetic acid (EDTA), ammonium acetate and Phenylmethanesolfonyl fluoride (PMSF) and the other chemicals used for protein extraction and separation were supplied with analytical grade. Double distilled water (ddH_2_O) was used for preparing all solutions and buffers.

### Plant Material and Growth Conditions

According to Talei et al. [48], the seeds of the salt-tolerant accession of *A. paniculata* (11329) were collected from the Agro Gene Bank, Universiti Putra Malaysia. The seeds were germinated as described by Talei et al. [49], [50] and then incubated in a growth chamber under a light/dark regime of 14/10 h at 28–30°C, and relative humidity between 60–75%. The germinated seeds at initial leaf stage were transferred into the hydroponic system (Hoagland nutrient solution) to provide the plants with continual nutrient supplies. The seedlings were grown in a greenhouse at a controlled temperature of 20°C during the night and 28°C during the day. The 45-day-old seedlings were then treated with 0 (control) and 12 dS m^−1^ of sodium chloride solution. The sodium chloride was increased with a daily increment to accomplish the final concentrations of NaCl (12 dS m^−1^). Salt treatments above 12 dS m^−1^ would be lethal to the seedlings within a short period [51]; therefore, the 12 dS m^−1^ salt treatment was the maximum salinity used in this study. After 15 days of salinity exposure, fresh leaves and roots were collected and frozen immediately in liquid nitrogen to prevent protein degradation.

### Protein Sample Preparation and Quantification for 2-DE

Five grams of the fresh and fully expanded leaves from 5–10 healthy 45-day-old plants of each accession were collected and thoroughly washed with deionised water, and were then frozen in liquid nitrogen. Two grams of the frozen leaf and root tissue samples of each control and salt-treated plants (accession 11329) were ground into a fine powder using the autoclaved and pre-chilled mortar and pestle, and then homogenized with 10 mL of extraction buffer (20 mM HEPES/KOH pH 7.5, 40 mM KCl, 1 mM EDTA, 10% (v/v) Glycerol and 1 mM PMSF) as described by Talei et al. [52].

The pellet was air dried and solubilised in a solubilization buffer containing 9.8 M Urea, 4% CHAPS, 65 mM DTT and 200 mM Tris base. The solubilised samples were dissolved in 500 µL rehydration buffer. The Bradford method [53] determined the total protein concentration by employing bovine serum albumin as a standard. The measurements were in triplicate, at 595 nm, using a Perkin Elmer (Lambda 25 UV/Vis) spectrophotometer.

### First and Second Dimension Protein Separation

The electrophoresis techniques used for proteome analysis were including the isoelectric focusing (IEF) and sodium dodecyl sulphate polyacrylamide gel electrophoresis (SDS-PAGE). Two-dimensional gel electrophoresis was carried out to resolve the protein mixtures as described by O'Farrell [54]. Approximately, 200 µg of the total proteins were extracted from the leaves and roots of *A. paniculata* seedlings, and then were solubilised in 20 µL solubilization buffer. The solubilised samples were placed in the separate tubes and incubated at room temperature for 30 min, and then mixed with 250 µL of rehydration buffer (9.8 M Urea, 2% CHAPS, 0.5% IPG buffer, 65 mM DTT and 0.1% Bromophenol blue). The total content of each tube was pipetted in a separate channel of the rehydration tray through an IPG strip gel (pH 4–7), in a bead-like manner. An IPG strip was located on each channel gel side down, ensuring that no bubbles were trapped underside the plastic side of the strip.

The strips were rehydrated at room temperature for 16 hours, and the strips were then transferred to an IEF focusing tray for the isoelectric focusing. The strip was placed on an IEF Mini-Protean II electrophoresis cell, and the first dimension (IEF) was performed with the focusing program of 250 V for two min, 500 V for 30 min, 1000 V for 1 h, 4000 V for 2 h, and a final focusing of 14000 volt-hour focusing step was at 4000 V Equilibration of the IPG strips were firstly performed with an equilibration buffer (6 M Urea, 50 mM Tris- HCl, pH 8.8, 2% SDS, 30% glycerol and 2% DTT (w/v), for 15 min. Immediately after the first equilibration step, the alkylation procedure was carried out by incubating each strip in the second equilibration buffer with 2.5% of iodoacetamide (IAA).

The second dimensional electrophoresis was implemented using the Laemmli method [55] on the 12% polyacrylamide gels using the Mini-Protean II electrophoresis cell. The electrophoretic separation was carried out using the running buffer (3% Tris base, 14.4% glycine, 1% SDS), for 90 minutes at 100 V.

### Staining and digitization of protein pattern

Prior to staining, the gels were fixed overnight in a fixative solution (30% Ethanol/10% Acetic acid) on a benchtop shaker SASTEC; ST-344 model, at 100 rpm. The gels were stained with 0.25% Coomassie Brilliant Blue R-250 in 40% (v/v) methanol and 7% (v/v) acetic acid for 1 h and de-stained with 40% (v/v) methanol and 7% (v/v) acetic acid until the backgrounds became clear. The de-stained gels were then transferred into the storage bags containing 25% methanol and were kept at 4°C. The gels were scanned and visualized using a densitometer. The scanned gels were saved as TIFF (Tagged Image File Format) images and exported to PDQuest 2-DE analysis software for further analysis.

To determine differences in protein expression, three gel images from each treatment containing the spots commonly present in all the three gels were analyzed using the PDQuest software. In brief, the software detects all the protein spots with background subtraction and then matches the spots of the control and treated samples. The detection parameters were determined by using reference spots covering small, faint and the largest spots on the master gel. During this process, additional filtering was also carried out to remove the horizontal and vertical streaks. At least three replicates of each gel slice belonging to each treatment were aligned to create a composite image containing only those proteins that are common to all three gels. The images from different treatments were aligned to create a super composite image using the matching tools. The 2-DE images of the salt-treated and control samples were matched with each other to determine the commonly induced proteins.

### Identification of the Functional Proteins

The selected induced, upregulated and downregulated leaf and root proteins of *A. paniculata* were trypsin digested and peptides were extracted according to the standard techniques [56]. The peptides were analyzed by automated matrix-associated laser desorption/ionization time-of-flight mass spectrometry (MALDI TOF MS) using a 5800 Proteomics Analyzer. Spectra were analyzed to identify the protein of interest using Mascot sequence matching software (Matrix Science) with Ludwig NR Database.

### Statistical analysis

Independent samples t-test was used for comparison of the representing proteins (repressed, induced, upregulated and downregulated proteins) in the control and salt-treated sample.
